# Practical Urinalysis and Urinary Diagnosis

**Published:** 1895-08

**Authors:** 


Practical Uranalysis and Urinary Diagnosis. A Manual for the Use of Physicians, Surgeons, and Students. By Charles W. Purdy, M. D., Queen's University; Fellow of the Royal College of Physicians and Surgeons, Kingston; Professor of Urology and Urinary Diagnosis at the Chicago Post-Graduate Medical School. Author of *Bright's Disease and Allied Affections of the Kidneys;* also of *Diabetes: Its Causes, Symptoms, and Treatment.* With numerous illustrations, including photo-engravings and colored plates. In one crown octavo volume, 360 pages, in extra cloth, $2.50 net. Philadelphia: The F. A. Davis Co., Publishers, 1914 and 1916 Cherry Street.

For this work we have only unqualified commendation. It contains the latest and best information upon the subject of the analysis of urine. In addition it contains an excellent account of the various kidney diseases and the method of diagnosing them. The renal diseases that have been considered in this book are as follows: Acute renal hyperæmia, passive renal hyperæmia, acute diffuse nephritis, chronic diffuse nephritis, acute and chronic interstitial nephritis, amyloid or waxy kidney, cystic disease of the kidney, renal tuberculosis, renal cancer, renal embolism, hydronephrosis, pyonephrosis, chronic pyelitis, and movable kidney.

These diseases are especially enumerated because, as every practitioner knows, there has been such continued controversy ever since the time of Bright, about 1828 or 1830, concerning the disease or assemblage of diseases to which his name was given. Every authority has given a different classification, a different set of names, and consequently different views of their nature. This is well seen by comparison of what is here said with the review of a book on Bright's Disease, by Dr. Robert Saundby, noticed in THE HOMŒOPATHIC PHYSICIAN for July, 1889, page 309.

Of course this book is not written with the prime object of discussing Bright's Disease. It is intended, instead, as its name sufficiently shows, to be a treatise upon the nature of the urine, the changes in it, and the method of detecting the changing elements, and the diagnosis of the urinary diseases. This mission it accomplishes very well, and it is, therefore, a fit successor of the old and justly celebrated work of Robertson.

With regard to the analysis of the urine, the author recommends as the best test for sugar in the urine, the formula of Prof. Walter S. Haines, of Chicago. This formula originally was as follows: Cupric sulphate, pure, 30 grains; pure glycerine, 4 drachms; caustic potassa in sticks, 3 drachms; distilled water, 6 ounces. This formula has been simplified as follows: Copper sulphate, 30 grains; distilled water, ½ ounce; make a perfect solution; then add glycerine, pure, ½ ounce; mix thoroughly and add 5 ounces of liquor potassa. In testing with this solution take about 1 drachm and gently boil it in a test-tube. Then add not more than six or eight drops of the urine to be tested, and again boil carefully. If sugar be present, a copious yellowish red precipitate falls. The absence of this precipitate denotes the absence of sugar. This test is very reliable, does not change by keeping, and so is always ready for use. Elaborate directions are given for the quantitative testing for sugar also.

For albumin, the author gives all the usual tests, but points out the liabilities of error, especially in the test with heat and nitric acid. Thus the albumin may fail to be detected, because it combines with the acid to form a soluble compound called syntonine, which thus escapes the eye. Again, if the acid be insufficient and the phosphates be in excess, then a portion only of the phosphates will be acidified and the remainder will combine with the albumin, forming a soluble compound which cannot be precipitated. On the other hand, the precipitate may be not only albumin but globulin and mucin, which makes confusion. In view of these errors the author gives a modification of the old tests. This modification is especially useful where the percentage of albumin present is very small.

The author's method is as follows: Have at hand a solution of chloride of sodium, or table salt prepared by saturation and filtering, so that it is perfectly clear. Then add a little of this salt solution to the sample of urine to be tested, so as to raise the specific gravity. Into an ordinary test-tube pour in enough of the urine, so diluted, to fill it two-thirds full; add one or two drops of strong acetic acid, and boil the upper inch or so of the urine over a spirit lamp for about a minute. If albumin be present it will appear in the upper boiled portion of the urine as a more or less pronounced milk-like turbidity, while the lower or unboiled portion remains perfectly clear in strong contrast. "This test," says the author, "possesses all the sensitiveness of the heat and acid test for albumin and avoids the mucin reaction."

The other abnormal constituents of the urine are treated of, and the tests given with the same ability as in the two cases before cited, and so it would appear that this work fills every requirement of the earnest student of the disorders of the urinary tract.

